# The Elias University Hospital Approach: A Visual Guide to Ultrasound-Guided Botulinum Toxin Injection in Spasticity, Part IV—Distal Lower Limb Muscles

**DOI:** 10.3390/toxins17100508

**Published:** 2025-10-16

**Authors:** Marius Nicolae Popescu, Claudiu Căpeț, Cristina Popescu, Mihai Berteanu

**Affiliations:** 1 Department of Physical and Rehabilitation Medicine, Elias Emergency University Hospital, Carol Davila University of Medicine and Pharmacy, 020021 Bucharest, Romania; marius.popescu@umfcd.ro (M.N.P.); mberteanu@gmail.com (M.B.); 2 Clinic of Physical and Rehabilitation Medicine, Elias Emergency University Hospital, 011461 Bucharest, Romania; claudiu.capet@gmail.com; 3 Department of Oncologic Dermatology, Elias Emergency University Hospital, Carol Davila University of Medicine and Pharmacy, 020021 Bucharest, Romania

**Keywords:** post-stroke spasticity, botulinum toxin-A injections, ultrasound-guided therapy, distal lower limb muscles, musculoskeletal ultrasound

## Abstract

Spasticity of the distal lower limb substantially impairs stance, gait, and quality of life in patients with upper motor neuron lesions. Although ultrasound-guided botulinum toxin A (BoNT-A) injections are increasingly employed, structured, muscle-specific visual guidance for the distal lower limb remains limited. This study provides a comprehensive guide for ultrasound-guided BoNT-A injections across ten key distal lower limb muscles: gastrocnemius, soleus, tibialis posterior, flexor hallucis longus, flexor digitorum longus, tibialis anterior, extensor hallucis longus, flexor digitorum brevis, flexor hallucis brevis, and extensor digitorum longus. For each muscle, we present (1) Anatomical positioning relative to osseous landmarks; (2) Sonographic identification cues and dynamic features; (3) Zones of intramuscular neural arborization optimal for injection; (4) Practical injection protocols derived from literature and clinical experience. High-resolution ultrasound images and dynamic videos illustrate real-life muscle behavior and guide injection site selection. This guide facilitates precise targeting by correlating sonographic signs with optimal injection zones, addresses common spastic patterns—including equinus, varus, claw toe, and hallux deformities—and integrates fascial anatomy with motor-point mapping. This article completes the Elias University Hospital visual series, providing clinicians with a unified framework for effective spasticity management to improve gait, posture, and patient autonomy.

## 1. Introduction

Lower-limb spasticity frequently presents with gait-limiting deformities—including equinus, varus, claw-foot and toe malalignments—that markedly compromise ambulation, balance, postural stability and quality of life in patients with upper-motor-neuron conditions such as stroke, cerebral palsy and traumatic brain injury [1]. Established sonographic atlases (for example, Jost’s Atlas of Botulinum Toxin Injection and Alter’s Ultrasound-Guided Chemodenervation Procedures) and procedural ultrasound-BoNT guides (for example, EUROMUSCULUS/USPRM) provide essential anatomic guidance and standard injection sites [2,3,4]. However, clinicians also require actionable, muscle-specific guidance that integrates evidence on intramuscular neural arborization with reproducible sonographic landmarks and explicit dynamic evaluation protocols—elements that are seldom presented together in a single, workflow-oriented resource.

This fourth part continues and expands the systematic Elias University Hospital (EUH) approach developed in Parts I–III, and reflects our day-to-day clinical experience managing lower and upper limb spasticity [5,6,7,8]. We present a systematic, ultrasound-based approach for identifying and injecting key distal lower-limb muscles. For each muscle we specify its clinical significance, reproducible sonographic landmarks, characteristic dynamic behaviour, mapped zones of intramuscular neural arborization, and ultrasound-guided injection techniques, integrating evidence from the literature with practical insights derived from our routine EUH clinical practice.

Unique to this series is the integration of (i) motor-endplate/arborization synthesis with sonographic targeting rules; (ii) muscle-specific dynamic assessment protocols that show how activation is best demonstrated and isolated; (iii) concise “zones of interest” that triangulate maximal sonographic bulk with reported arborization; and (iv) workflow tools—a clinical decision table and four short annotated videos—designed to operationalize these elements in routine practice. Together, these features deliver a reproducible, clinically oriented roadmap for the gastrocnemius, soleus, tibialis posterior, flexor hallucis longus, flexor digitorum longus, tibialis anterior, extensor hallucis longus, flexor digitorum brevis, flexor hallucis brevis and extensor digitorum longus, complementing and extending conventional atlas resources.

## 2. General Methodology

Ultrasound-guided botulinum toxin injections were performed using a GE Venue Go ultrasound system equipped with a 12L linear transducer. Patient positioning was adjusted according to the examined anatomical region; the standard positions for each site are illustrated in the clinical photographs that accompany the corresponding ultrasound images.

## 3. Distal Lower Limb Muscles Implicated in Post-Stroke Spasticity

### 3.1. Gastrocnemius Muscles (Gastroc)

#### 3.1.1. Overview

The gastrocnemius muscle (Gastroc) pair is commonly targeted in spastic lower-limb patterns involving resistance to passive movement during ankle dorsiflexion, as well as during knee extension [9].

#### 3.1.2. Lateral Head of Gastrocnemius (LG)

##### Ultrasound Identification

In our clinical practice, the LG is identified using musculoskeletal ultrasound by placing the transducer transversely on the posterior aspect of the leg, approximately 8 cm distal to the popliteal crease, on the lateral side. Superficial to the fibular cortex, two muscle masses can be visualized: the deeper soleus and the overlying lateral gastrocnemius

##### Key Ultrasound Landmarks

**Figure 1:**
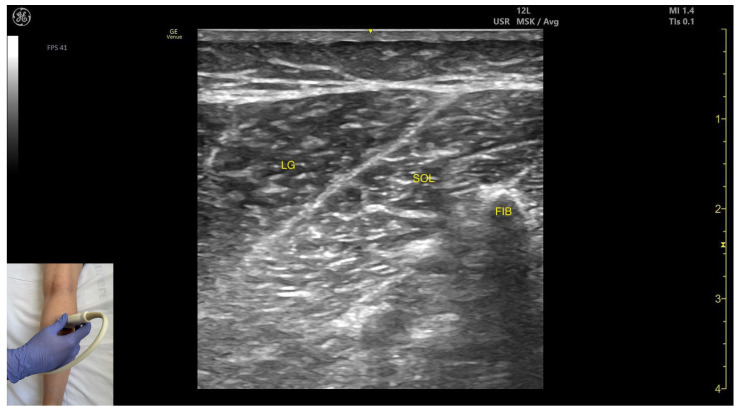
Ultrasound anatomy of the lateral head of gastrocnemius with key landmarks: LG—lateral head of gastrocnemius; SOL—soleus; FIB—fibula.

The key ultrasound landmarks include the following (Figure 1) [9,10,11,12,13,14,15,16]:

- Muscle position: It is the most superficial and prominent muscle in the posterior compartment of the leg, and one of the largest in the region. The soleus—and, if present, the plantaris—is located deep to it.
- External fascia: It presents a well-defined fascia separating it from the subcutaneous plane, soleus, and plantaris muscle/tendon, during BoNT-A injection.
- Dynamic evaluation: During dynamic evaluation, scanning toward the ankle joint, the lateral head of the gastrocnemius progressively decreases and eventually disappears while the soleus increases in thickness; distally, both merge into the Achilles tendon with the soleus (and plantaris, if present). On medial scanning the intersection of the lateral and medial gastrocnemius heads is visualized—the medial sural cutaneous nerve typically courses between these heads. Because the gastrocnemius is a biarticular muscle crossing both the knee and ankle joints, it is assessed with the knee extended to place the muscle on maximal stretch and thereby reveal its contribution to ankle plantarflexion. Muscle contraction is visible during plantar flexion of the foot at the ankle joint, as well as during knee flexion.

#### 3.1.3. Medial Head of Gastrocnemius (MG)

##### Ultrasound Identification

In our clinical practice, the MG can be identified using musculoskeletal ultrasound by placing the transducer transversely on the posterior aspect of the leg, approximately 8 cm distal to the popliteal crease, on the medial side. Superficial to the tibial cortex, the deeper soleus and the overlying medial gastrocnemius are identified.

##### Key Ultrasound Landmarks

**Figure 2:**
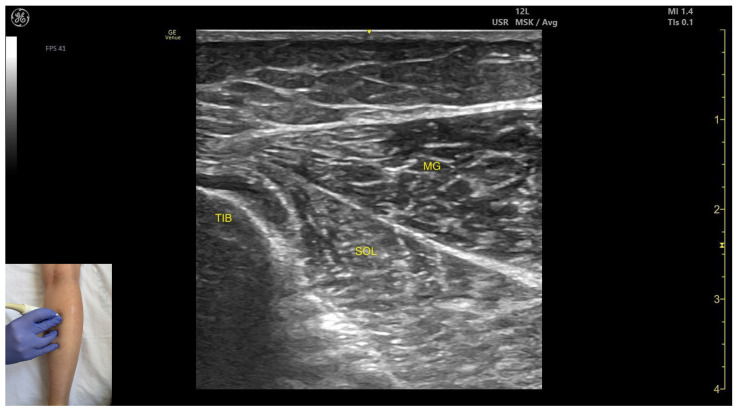
Ultrasound anatomy of the medial head of gastrocnemius with key landmarks: TIB—tibia; MG—medial head of gastrocnemius; SOL—soleus.

The key ultrasound features include the following (Figure 2) [9,10,11,12,14,15,16]:

- Muscle position: It is the most superficial and prominent, and of the largest muscles in the posterior compartment of the leg. The soleus—and if present, the plantaris—is located deep to it.
- Muscle morphology: The medial head of the gastrocnemius is larger and extends more distally than the lateral head of the gastrocnemius.
- External fascia: It presents a well-defined fascia separating it from the subcutaneous plane, soleus, and plantaris muscle/tendon aiding in safe BoNT-A injection.
- Dynamic evaluation: During dynamic evaluation, scanning toward the ankle joint reveals the medial head of the gastrocnemius progressively decreases and eventually disappears while the soleus increases in thickness; further distally, both converge to form the calcaneal (Achilles) tendon, often with the plantaris tendon interposed when present. On lateral scanning the intersection of the medial and lateral gastrocnemius heads is visualized—the medial sural cutaneous nerve typically courses between these heads. The gastrocnemius, spanning both the knee and ankle joints as a biarticular muscle, is evaluated with the knee fully extended to place it under maximal longitudinal tension, thereby accentuating its functional role in ankle plantarflexion. Contraction is visible during plantar flexion of the foot at the ankle joint, as well as during knee flexion.

#### 3.1.4. Clinical Implications & Injection Strategy

The gastrocnemii are commonly implicated in spastic patterns such as equinus foot and equinovarus [2,17].

According to Gracies et al., local injections of botulinum toxin into the gastrocnemius and the hamstring muscle groups attenuate co-contraction and result in functional improvements in cerebral palsy [18].

Picelli et al. compared clinical outcomes of gastrocnemius BoNT injections performed under ultrasound guidance, electrical stimulation, or blind (manual) needle placement and reported a significantly superior effect with ultrasound guidance, with greater reductions in the Modified Ashworth Scale (MAS) and larger gains in passive range of motion (PROM) at the ankle joint [19].

In the paediatric population with cerebral palsy, focal BoNT injections into the gastrocnemius improved the MAS for the foot and produced improvements in Gross Motor Function Measure (GMFM) scores at four weeks post-injection [20].

A 2018 study reported that gastrocnemius injections resulted in a decreased pennation angle and an increased fascicle length; these changes were attributed to reduction in spasticity and subsequent structural remodeling of muscle fibers [21].

The regions with the highest intramuscular neural arborization are located at approximately four-fifths of the muscle length for the lateral gastrocnemius and at approximately three-quarters for the medial gastrocnemius, when measured along the reference line joining the intercondylar line to the intermalleolar line [22].

In our clinical practice, the preferred injection site for BoNT-A is at the point of maximum muscle thickness identified via musculoskeletal US. For the medial head, this corresponds to the transducer placed transversely over the posterior aspect of the leg, approximately 8 cm distal to the popliteal crease in the medial portion; for the lateral head the transducer is placed in the same transverse orientation, approximately 8 cm distal to the popliteal crease, in the lateral portion.

A potential source of error at this level is the presence of the plantaris muscle and its slender tendon, which courses between the gastrocnemius and soleus and can be mistaken for a separate muscular or tendinous structure [23].

Musculoskeletal ultrasound provides direct visualization of fascial planes, underlying muscle architecture, and adjacent neurovascular structures. It enhances procedural precision, reduces the risk of nerve injury, and ensures accurate delivery of BoNT-A into the intended muscular compartment. This technique has proven especially valuable in patients with anatomical variability due to chronic spasticity or postural deformities.

### 3.2. Soleus (SOL)

#### 3.2.1. Overview

The soleus (SOL) is a muscle commonly targeted in spasticity patterns of the lower limb, involving resistance to passive movement during dorsiflexion of the ankle joint [9].

#### 3.2.2. Ultrasound Identification

In our clinical practice, the SOL can be identified using musculoskeletal ultrasound with the transducer placed transversely on the posterior aspect of the leg, approximately 8 cm distal to the popliteal crease on the midline. Superficial to the tibial cortex, two muscle masses are visualized: the soleus (deep) and the gastrocnemius (superficial).

#### 3.2.3. Key Ultrasound Landmarks:

**Figure 3:**
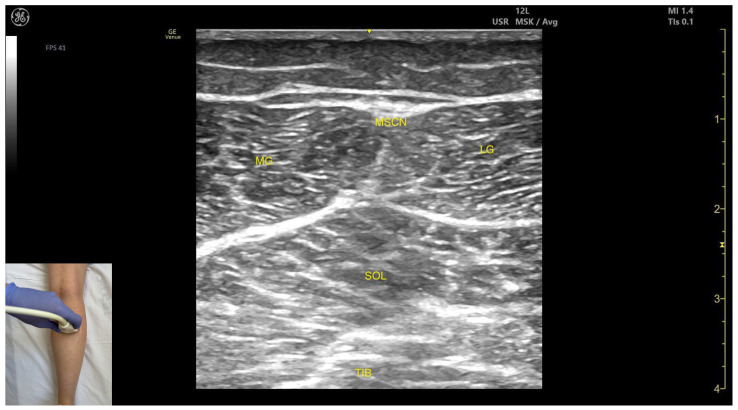
Ultrasound anatomy of the soleus: MG—medial head of the gastrocnemius; MSCN—medial sural cutaneous nerve; LG—medial head of gastrocnemius; SOL—soleus; TIB—tibia.

The key ultrasound features include the following (Figure 3) [9,11,14,24,25,26]:

- Muscle morphology: It appears as a flat muscle in the posterior compartment of the leg.
- Muscle position: At this level, the soleus lies deep to the intersection of the gastrocnemius heads, within which the medial sural cutaneous nerve courses.
- External fascia: A pronounced fascia separates it from the subcutaneous plane, gastrocnemius muscles, and the plantaris muscle/tendon (if present), which is relevant during BoNT-A injection.
- Dynamic evaluation: During dynamic evaluation, scanning laterally reveals enlargement of the lateral gastrocnemius head, and deep to the soleus, the neurovascular bundle emerges, comprising the posterior tibial artery, the two posterior tibial veins, and the posterior tibial nerve. Medial scanning highlights enlargement of the medial head of the gastrocnemius (MG), while the neurovascular bundle maintains its anatomical course. The soleus, a monoarticular muscle acting exclusively at the ankle joint, is optimally assessed with the knee flexed; this positioning diminishes gastrocnemius involvement, allowing for isolated evaluation of soleus contractile activity via EMG or dynamic ultrasound imaging. Muscle contraction is visible during plantar flexion maneuvers of the foot at the ankle joint.

#### 3.2.4. Clinical Implications and Injection Strategy

Spasticity of the SOL muscle contributes to equinus foot, equinovarus, and drop-foot deformities [2,17,27,28]. Local BoNT-A injections into the soleus have been shown to reduce spasticity and significantly improve walking speed, stride length, premature muscle activation, and stance symmetry [17,29,30]. According to Boyaci et al., children with cerebral palsy who received combined gastrocnemius and soleus injections—using a lower dose (1.5 U/kg vs. the 2.6 U/kg typically recommended)—demonstrated significant increases in muscle thickness, although changes in the gastrocnemius may also be influenced by intensive strengthening, stretching, and ambulatory training following injection [31]. The zones with the highest density of intramuscular nerve arborizations in the soleus are located at three-fifths of its length, both medially and laterally, along the reference line connecting the medial malleolus to the proximal border of the medial tibial condyle [22].

Using musculoskeletal ultrasound to guide injections into the soleus allows for precise placement within the target muscle and minimizes the risk of neurovascular injury. In our clinical practice, preferred injection sites correspond to the points of maximum muscle thickness identified via musculoskeletal ultrasound: the transducer is placed transversely on the posterior leg approximately 8 cm distal to the popliteal crease—medially for the first injection site and laterally for the second. A common source of error at this level is the presence of the plantaris muscle/tendon, which lies between the gastrocnemius and soleus.

### 3.3. Tibialis Posterior (TP)

#### 3.3.1. Overview

The posterior tibialis (TP) is a muscle targeted in lower-limb spasticity patterns that involve resistance to passive movement during ankle eversion and plantar dorsiflexion [9].

#### 3.3.2. Anterior Window

##### Ultrasound Identification

In our clinical practice, the TP muscle can be identified using musculoskeletal ultrasound by placing the transducer transversely on the anterior aspect of the leg, approximately 10 cm distal to the knee joint, specifically in the lateral portion. Superficial to the tibial and fibular cortices, two muscle masses are visualized: the tibialis anterior medially and the extensor digitorum longus laterally. The interosseous membrane is seen between the two bones, and deep to this membrane lies the tibialis posterior.

##### Key Ultrasound Landmarks

**Figure 4:**
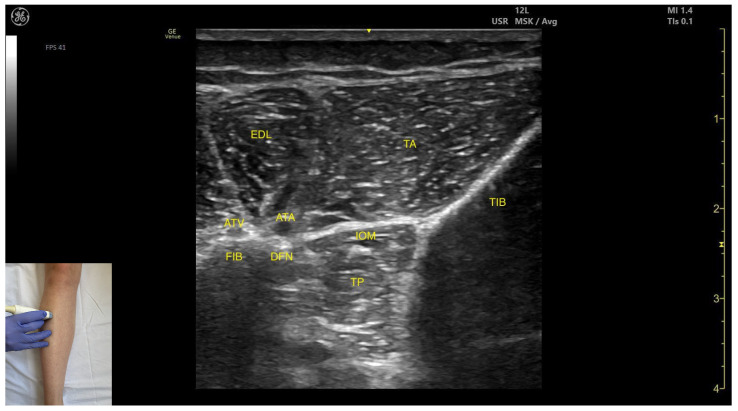
Ultrasound anatomy of the tibialis posterior—anterior window: ATV—anterior tibial vein; ATA—anterior tibial artery; FIB—fibula; DFN—deep fibular nerve; EDL—extensor digitorum longus; TA—tibialis anterior; IOM—interosseous membrane; TP—tibialis posterior; TIB—tibia.

The key ultrasound features include the following (Figure 4) [9,14]:

- Muscle position: It is the deepest muscle in the posterior compartment of the leg.
- Neurovascular bundle: Superficial to the interosseous membrane lies the neurovascular bundle composed of the deep peroneal nerve, anterior tibial artery, and anterior tibial vein.
- External fascia: TP has a pronounced fascia that separates it from the tibialis anterior and extensor digitorum longus during BoNT-A injection.
- Dynamic evaluation: During dynamic evaluation muscle contraction is visible during inversion and plantar flexion of the ankle joint.

#### 3.3.3. Medial Window

##### Ultrasound Identification

In our clinical practice, the TP muscle can be identified using musculoskeletal ultrasound by placing the transducer transversely at the distal third of the leg, specifically in the medial portion. At this level, the cortical surfaces of the tibia and fibula are visualized, with the TP located deep to them. Superficial and medial to the TP lies the flexor digitorum longus, while the flexor hallucis longus is positioned superficially and laterally. The most superficial muscle in this region is the soleus.

##### Key Ultrasound Landmarks

**Figure 5:**
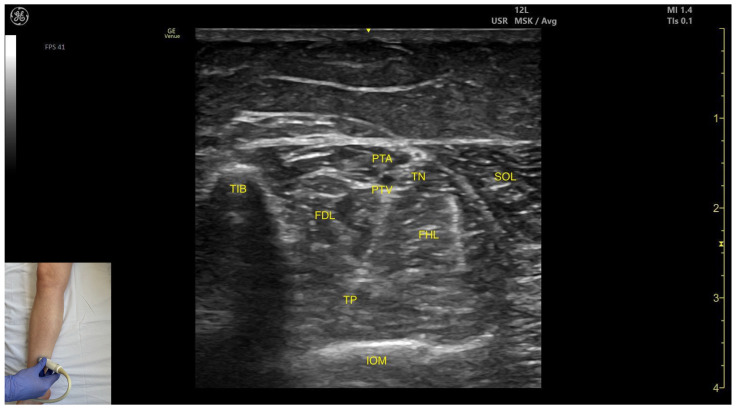
Ultrasound anatomy of the tibialis posterior—medial window: TIB—tibia; FDL—flexor digitorum longus; TP—tibialis posterior; PTA—posterior tibial artery; PTV—posterior tibial vein; TN—tibial nerve; FHL—flexor hallucis longus; IOM—interosseous membrane; SOL—soleus.

The key ultrasound landmarks include the following (Figure 5) [9,14]:

- Muscle position: The tibialis posterior is the deepest muscle in the posterior compartment of the leg. It is bordered superficially and medially by the flexor digitorum longus and superficially and laterally by the flexor hallucis longus. The interosseous membrane lies deep to the TP.
- Neurovascular bundle: Within the intermuscular fascial plane between the flexor digitorum longus, flexor hallucis longus, and soleus, the tibial nerve, posterior tibial artery, and posterior tibial vein are situated.
- External fascia: The TP does not have a well-defined fascia that separates it from flexor digitorum longus and flexor hallucis longus, during BoNT-A injection.
- Dynamic evaluation: Muscle contraction becomes evident during ankle inversion and plantar flexion at the ankle joint.

#### 3.3.4. Posterior Window

##### Ultrasound Identification

In our clinical practice, the TP muscle can be identified using musculoskeletal ultrasound by placing the transducer transversely on the distal third of the posterior leg. Superficial to the cortical surfaces of the tibia and fibula, the TP is visualized. Superficial and lateral to the TP lies the flexor hallucis longus, whereas the flexor digitorum longus is superficial and medial. The most superficial muscle at this level is the soleus.

##### Key Ultrasound Landmarks

**Figure 6:**
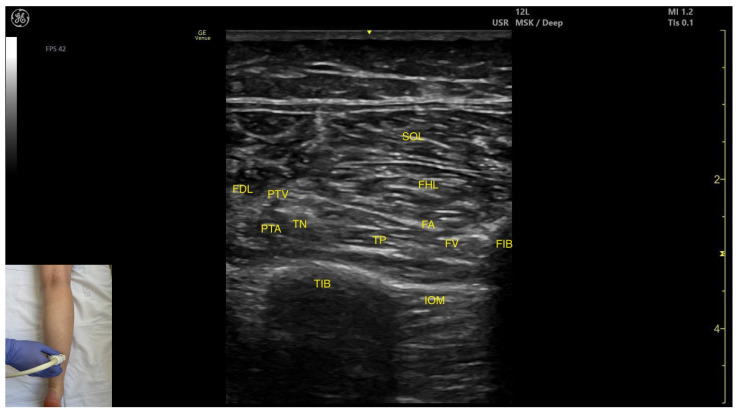
Ultrasound anatomy of the tibialis posterior—posterior window: FDL—flexor digitorum longus; PTA—posterior tibial artery; PTV—posterior tibial vein; TN—tibial nerve; TIB—tibia; TP—tibialis posterior; SOL—soleus; FHL—flexor hallucis longus; FA—fibular artery; FV—fibular vein; IOM—interosseous membrane; FIB—fibula.

The key ultrasound landmarks include the following (Figure 6) [9,14]:

- Muscle position: The tibialis posterior is the deepest muscle in the posterior compartment of the leg. It is bordered superficially and medially by the flexor digitorum longus, and superficially and laterally by the flexor hallucis longus. The interosseous membrane is located deep to the TP.
- Neurovascular bundle: In the intermuscular fascia between the TP and the FHL run the fibular artery and vein. Additionally, in the intermuscular fascia formed by the soleus, FDL and FHL are the tibial nerve, the posterior tibial artery and the posterior tibial vein.
- External fascia: The TP has pronounced fascia that separates it from the flexor digotorum longus and the flexor hallucis longus, which is relevant for BoNT-A injection.
- Dynamic evaluation: Muscle contraction is visible during ankle inversion and plantar flexion at the ankle joint.

#### 3.3.5. Clinical Implications and Injection Strategy

Spasticity of the posterior tibialis contributes to equinus foot and equinovarus deformities [2,17,27,32,33]. The highest density of intramuscular nerve arborizations is most concentrated at two key zones: 10–40% and 70–80% along the line from the medial proximal tibial articular margin to the distal medial malleolus, as identified by Lee et al.; and 70–80% along the line between the lateral malleolus and fibular head, as reported by Yi et al. [33,34,35].

In our clinical practice, the preferred injection site for BoNT-A is at the point of maximum muscle thickness identified via musculoskeletal US. For the TP, the first injection site is identified with the transducer placed transversely on the anterior aspect of the leg approximately 10 cm distal to the knee joint on the lateral side. The second site is obtained with the transducer placed transversely either on the distal third of the medial leg or on the distal third of the posterior leg. This ultrasound-guided approach enables precise administration of BoNT-A directly into the posterior tibialis while significantly reducing the risk of neurovascular injury.

### 3.4. Flexor Hallucis Longus (FHL)

#### 3.4.1. Overview

The flexor hallucis longus (FHL) is a muscle targeted in lower-limb spasticity patterns involving resistance to passive movement during hallux extension at the metatarsophalangeal and interphalangeal joints, and dorsiflexion at the ankle joint [36].

#### 3.4.2. Ultrasound Identification

In our clinical practice, the FHL muscle can be identified using musculoskeletal ultrasound by placing the transducer transversely on the distal third of the posterior leg, in the lateral portion. Superficial and medial to the fibular cortex, the FHL is visualized.

#### 3.4.3. Key Ultrasound Landmarks

**Figure 7:**
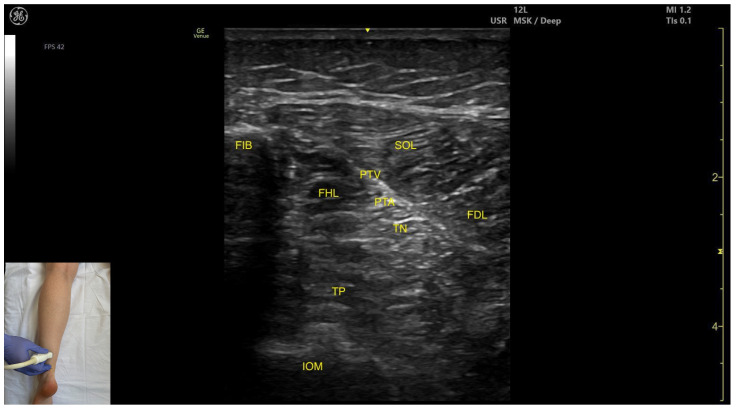
Ultrasound anatomy of the flexor hallucis longus: FIB—fibula; FHL—flexor hallucis longus; TP—tibialis posterior; IOM—interosseous membrane; PTA—posterior tibial artery; PTV—posterior tibial vein; TN—tibial nerve; SOL—soleus; FDL—flexor digitorum longus.

The key ultrasound landmarks include the following (Figure 7) [36]:

- Muscle morphology: It is a strong, unipennate muscle. In our clinical practice it is referred to as the “shark tail”
- Muscle position: It is located superficial to the fibular cortex. The SOL lies superficial to the FHL. Deep and medial to it lies the tibialis posterior (TP) and, deep to TP, the interosseous membrane. Medial to this region is the flexor digitorum longus (FDL). At this level, the usual anatomical relationship of the FHL and FDL is paradoxically reversed.
- Neurovascular bundle: The tibial nerve, posterior tibial artery, and posterior tibial vein lie within the intermuscular fascia between tibialis posterior, SOL, FDL.
- External fascia: The FHL has a well-defined fascia that separates it from soleus, aiding in safe BoNT-A injection. It does not have a pronounced fascia that separates it from the tibialis posterior and flexor digitorum longus.
- Dynamic evaluation: During dynamic evaluation, scanning proximally toward the knee joint, the FHL muscle thickness decreases, and it disappears from view, while the soleus thickens and the lateral head of the gastrocnemius appears superficially (Video S1). Muscle contraction is seen during flexion of the hallux at the metatarsophalangeal and interphalangeal joints, as well as during plantar flexion at the ankle joint.

#### 3.4.4. Clinical Implications and Injection Strategy

Spasticity of the FHL is characterized by a fixed or discontinuous flexion of the hallux. It contributes to the equinus/equinovarus gait pattern alongside the gastrocnemius, soleus, posterior tibialis, flexor hallucis brevis, and extensor hallucis brevis [17,27]. It is also implicated in claw-toe deformity in combination with the flexor digitorum longus [27,37].

Beyond reducing hallux spasticity, BoNT-A injections into the FHL are used to alleviate muscle spasms and pain during gait [38]. The densest intramuscular neural arborization zones of the FHL are located at approximately 30–40% and 60–70% along the line connecting the most prominent point of the lateral malleolus to the fibular head [34].

Musculoskeletal US guidance enables precise targeting of the injection site and helps avoid neurovascular injury. In our clinical practice, the preferred injection zones for BoNT-A injections are at the points of maximum muscle thickness, identified via musculoskeletal US, with the transducer placed at the distal third of the posterior leg for the first site and at the middle third for the second site.

### 3.5. Flexor Digitorum Longus (FDL)

#### 3.5.1. Overview

The flexor digitorum longus (FDL) is a muscle targeted in spasticity patterns of the lower limb, which include resistance to passive extension of toes II–V at the metatarsophalangeal and interphalangeal joints and dorsiflexion of the ankle [36].

#### 3.5.2. Ultrasound Identification

In our clinical practice, the FDL can be identified using musculoskeletal ultrasound by placing the transducer transversely on the distal third of the posterior leg in the medial portion. Superficial and lateral to the tibial cortex, the flexor digitorum longus muscle is visualized.

#### 3.5.3. Key Ultrasound Landmarks

**Figure 8:**
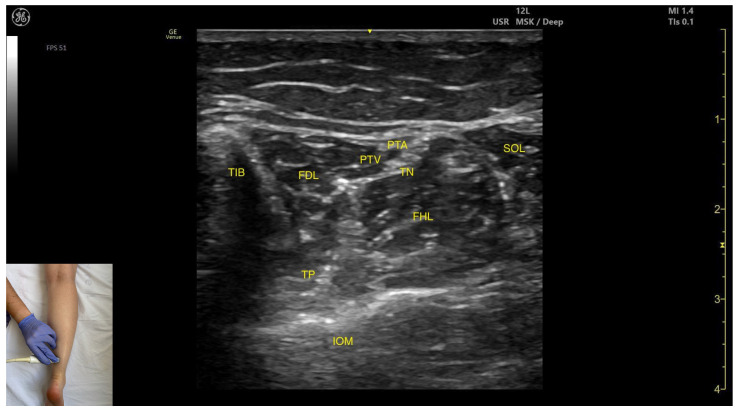
Ultrasound anatomy of the flexor digitorum longus: TIB—tibia; FDL—flexor digitorum longus; TP—tibialis posterior; IOM—interosseous membrane; PTA—posterior tibial artery; PTV—posterior tibial nerve; FHL—flexor hallucis longus; SOL—soleus; TN—tibial nerve.

The key ultrasound landmarks include the following (Figure 8) [9,14,36]:

- Muscle position: It is located in the medial compartment of the posterior leg. Superficially, it is covered by the soleus; lateral to the FDL lies the flexor hallucis longus; deep and lateral to it lie the TP with the interosseous membrane located beneath it. At this level, the flexor digitorum longus and flexor hallucis longus are paradoxically reversed in position relative to their usual anatomical course.
- Neurovascular bundle: Within the intermuscular fascia between the flexor digitorum longus, flexor hallucis longus, and soleus are the tibial nerve, posterior tibial artery, and posterior tibial veins.
- External fascia: The FDL has a well-defined fascia that separates it from the soleus, which is relevant during BoNT-A injection. It does not have a pronounced fascia that separates it from the flexor hallucis longus and tibialis posterior.
- Dynamic evaluation: During dynamic evaluation, scanning proximally toward the knee joint, the flexor digitorum longus decreases in thickness and disappears from view, while the soleus increases in size and the medial head of the gastrocnemius appears superficial to the soleus (Video S2). Muscle contraction is visible during flexion of toes II–V (at metatarsophalangeal, proximal and distal interphalangeal joints) and ankle plantar flexion.

#### 3.5.4. Clinical Implications and Injection Strategy

The FDL is involved in the equinus/equinovarus spasticity pattern [17,27]. FDL spasticity contributes to the characteristic “claw toes” deformity. Beyond reducing spasticity, BoNT-A injections have also been shown to relieve spastic foot pain [39]. The region with the highest density of intramuscular nerve arborization is located at 40–50% along the reference line from the most prominent point of the lateral malleolus (0%) to the fibular head (100%) [34].

Musculoskeletal ultrasound guidance ensures accurate placement of the toxin at the target site and helps prevent the damage to nearby neurovascular structures. In our clinical practice, the preferred injection site corresponds to the area of maximal muscle thickness detected via musculoskeletal US, with the transducer placed transversely at the mid-level of the posterior aspect of the leg in the medial portion.

### 3.6. Tibialis Anterior (TA)

#### 3.6.1. Overview

The tibialis anterior (TA) is targeted in lower-limb spasticity patterns, which include passive resistance during plantar flexion and plantar eversion of the ankle joint [36].

#### 3.6.2. Ultrasound Identification

In our clinical practice, the TA can be identified using musculoskeletal ultrasound by placing the transducer transversely on the proximal third of the anterior aspect of the leg, in the lateral portion. Superficial and lateral to the tibial cortex, the tibialis anterior muscle is visualized.

#### 3.6.3. Key Ultrasound Landmarks

**Figure 9:**
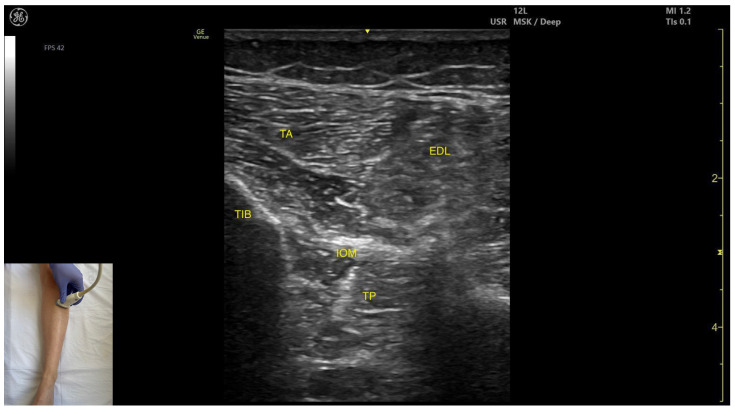
Ultrasound anatomy of the tibialis anterior: TIB—tibia; TA—tibialis anterior; IOM—interosseous membrane; TP—tibialis posterior; EDL—extensor digitorum longus.

The key ultrasound landmarks include the following (Figure 9) [14,36]:

- Muscle position: It appears as a superficial muscle mass. Deep to the TA lies the interosseous membrane separating it from the tibialis posterior; the extensor digitorum longus lies lateral to the TA.
- External fascia: The TA does not feature a pronounced fascia that separates it from extensor digitorum longus, during BoNT-A injection.
- Dynamic evaluation: During dynamic evaluation, scanning distally toward the ankle, the tibialis anterior decreases in thickness as it transitions into its tendon. Muscle contraction is seen during plantar dorsiflexion and inversion of the ankle joint.

#### 3.6.4. Clinical Implications and Injection Strategy

The tibialis anterior is commonly involved in spastic equinus patterns of the lower limb [27].

The tibialis anterior contributes to the spastic varus deformity of the ankle and foot, but its role in the equinovarus pattern is minimal or negligible [32].

The highest density of intramuscular neural arborizations in the tibialis anterior is located between 70 and 80% along the reference line connecting the lateral malleolus to the fibular head [40]. In our clinical practice, the preferred injection site corresponds to the area of maximal muscle thickness detected via musculoskeletal US, with the transducer placed transversely on the proximal third of the anterior aspect of the leg, in the lateral portion.

### 3.7. Extensor Hallucis Longus (EHL)

#### 3.7.1. Overview

The extensor hallucis longus (EHL) is a muscle involved in spasticity patterns of the lower limb, which include resistance to passive movement during hallux flexion at the metatarsophalangeal and interphalangeal joints, as well as resistance to plantar flexion at the ankle joint [41].

#### 3.7.2. Ultrasound Identification

In our clinical practice, the EHL can be identified using musculoskeletal ultrasound by placing the transducer transversely on the distal third of the anterior aspect of the leg, in lateral portion. Lateral to the tibial cortex, both the EHL and the extensor digitorum longus can be identified.

#### 3.7.3. Key Ultrasound Landmarks

**Figure 10:**
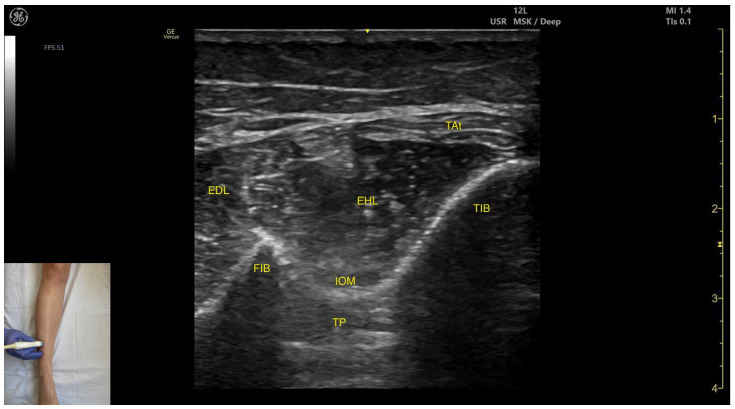
Ultrasound anatomy of the extensor hallucis longus: EDL—extensor digitorum longus; FIB—fibula; EHL—extensor hallucis longus; IOM—interosseous membrane; TP—tibialis posterior; TAt—tibialis anterior tendon; TIB—tibia.

The key ultrasound landmarks include the following (Figure 10) [36,38,41,42]:

- Muscle morphology: It is an unipennate muscle.
- Muscle position: It appears as a superficial muscle mass at this level. Laterally, the extensor digitorum longus (EDL) is visualized; superficially and laterally is the tendinous portion of the tibialis anterior (TA). Deep to the EHL lie the interosseous membrane and the tibialis posterior (TP).
- External fascia: The ELH does not have a well-defined fascia that separates it from the subcutaneous plane, the extensor digitorum longus, tibialis anterior. The interosseous membrane separates the EHL from the tibialis posterior, during BoNT-A injection.
- Dynamic evaluation: During dynamic evaluation, scanning proximally toward the knee joint shows a reduction in EHL muscle thickness with a concurrent increase in tibialis muscle thickness (Video S3). Muscle contraction is visible during hallux extension at the metatarsophalangeal and interphalangeal joints and during dorsiflexion at the ankle joint. It is clinically relevant in relation to the Babinski sign, often referred to as the “hitchhiker’s great toe,” which is characterized by dorsiflexion of the hallux in response to stimulation of the plantar surface of the foot.

#### 3.7.4. Clinical Implications and Injection Strategy

The extensor hallucis longus (EHL) is involved in spastic equinus and equinovarus patterns [27]. It also contributes to spastic hitchhiker’s toe and hallux claw toe deformities [38,43,44]. In such cases, patients may experience difficulty with shoe wear, pain, and gait instability [17,38]. Walking may be further impaired due to forefoot ground contact and weight bearing along the lateral border of the foot, which predispose to cutaneous complications such as pressure points, ulcerations, and callus formation [43].

The EHL is also implicated in hallux valgus deformity, together with the transverse and oblique heads of the adductor hallucis and the flexor hallucis brevis. Local botulinum toxin injections targeting these muscles have been shown to reduce hallux valgus–related pain for up to six months and to prevent further progression of the hallux valgus angle [45].

According to a 2020 study, in striatal toe deformity spasticity of the EHL develops progressively as a compensatory mechanism to enhance foot stability during the stance phase, thereby counteracting plantar flexor spasticity and its mechanical consequence—equinus deformity [32].

The zone with the highest intramuscular nerve arborization in the EHL are broadly distributed along the muscle belly, located approximately 12 cm proximal to the bimalleolar line or at about 35% distally of the total lower limb length [46].

In our clinical practice, the preferred injection site corresponds to the area of maximal muscle thickness detected via musculoskeletal US, with the transducer placed transversely on the distal third of the anterior aspect of the leg, in lateral portion.

### 3.8. Flexor Digitorum Brevis (FDB)

#### 3.8.1. Overview

Flexor digitorum brevis (FDB) is a muscle targeted in spasticity patterns of the lower limb that involve resistance to passive movement during passive extension of toes II–V at the proximal interphalangeal joints [9].

#### 3.8.2. Ultrasound Identification

In our clinical practice, the FDB can be identified using musculoskeletal ultrasound by placing the transducer transversely at the mid-plantar aspect of the foot. Superficial and medial to the cuboid cortex and superficial and lateral to the navicular cortex the quadratus plantae and the flexor digitorum brevis—appearing as an oval superficial muscle mass—are visualized.

#### 3.8.3. Key Ultrasound Landmarks

**Figure 11:**
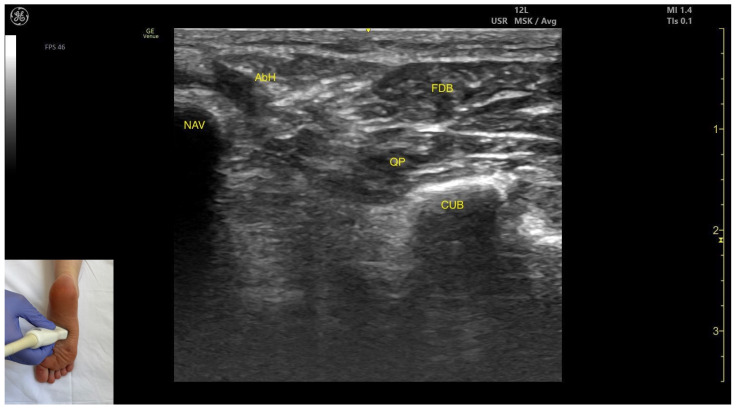
Ultrasound anatomy of the flexor digitorum brevis: NAV—navicular; AbH—abductor hallucis; FDB—flexor digitorum brevis; QP—quadratus plantae; CUB—cuboid.

The key ultrasound features include the following (Figure 11) [9,14]:

- Muscle position: It is a superficial plantar muscle layer. The quadratus plantae is located deep to the FDB. Medial to the FDB and quadratus plantae the abductor hallucis (AbH) is identified.
- External fascia: It features a prominent fascia separating it from the subcutaneous tissue, plantar aponeurosis and from the quadratus plantae, which is important during BoNT-A injection.
- Dynamic evaluation: During dynamic evaluation, scanning toward the calcaneous, a reduction in muscle thickness of the flexor digitorum brevis is observed. Muscle contraction is visible during flexion of toes II–V at the proximal interphalangeal joints (Video S4).

#### 3.8.4. Clinical Implications and Injection Strategy

The FDB contributes to spastic patterns such as hallux claw toe, claw foot, and equinus/equinovarus [17,37,44]. In our clinical practice, the preferred injection site corresponds to the area of maximal muscle thickness detected via musculoskeletal US, with the transducer placed transversely at the mid-plantar aspect of the foot. In the out-of-plane approach, the needle traverses the ultrasound beam and is seen only as a bright echogenic point, a technique that can facilitate access in narrow anatomical windows but limits visualization of the needle’s full trajectory [47]. In this case, we favor the in-plane approach, which enables continuous visualization of the needle and allows simultaneous targeting of FDB2, FDB3, FDB4, and FDB5 at their regions of maximal muscular thickness. This strategy reduces the number of punctures required and enhances patient acceptance of the procedure.

### 3.9. Flexor Hallucis Brevis (FHB)

#### 3.9.1. Overview

Flexor hallucis brevis (FHB) is a muscle targeted in spastic lower limb patterns, which include resistance to passive movement during extension of the hallux at the metatarsophalangeal joint [36].

#### 3.9.2. Ultrasound Identification

In our clinical practice, the FHB can be identified using musculoskeletal ultrasound by placing the transducer transversely approximately 1–2 cm proximal to the first metatarsophalangeal joint. Superficial to the cortex of the first metatarsal, the flexor hallucis brevis is visualized.

#### 3.9.3. Key Ultrasound Landmarks

**Figure 12:**
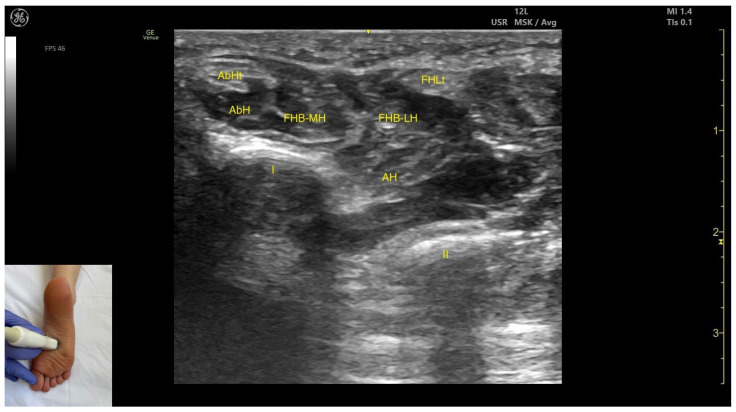
Ultrasound anatomy of the flexor hallucis brevis: AbH—abductor hallucis; AbHt—abductor hallucis tendon; FHB-MH—flexor hallucis brevis medial head; FHB-LH—flexor hallucis brevis—lateral head; FHLt—flexor hallucis longus tendon; I—first metatarsophalangeal joint; II—second metatarsal joint, AH—adductor hallucis.

The key ultrasound features include the following (Figure 12) [2,14,36]:

- Muscle position: It appears as a superficial muscle mass at this level. Medially, the abductor hallucis muscle and tendon are present; laterally, the tendon of the flexor hallucis longus is visible. Deep and medial to the FHB lies the adductor hallucis.
- Muscle morphology: It has two heads—medial and lateral—that can be targeted individually during BonT-A injection.
- External fascia: FHB has a pronounced fascia that separates it from the superficial tissue, the abductor hallucis muscle, and the tendon of the flexor hallucis longus, during BoNT-A injection.
- Dynamic evaluation: During dynamic evaluation, scanning distally toward the first metatarsophalangeal joint, a decrease in muscle thickness of the flexor hallucis brevis is observed along with its insertion on the medial and lateral sides of the proximal phalanx of the hallux. Muscle contraction is visible during dynamic evaluation when performing hallux flexion at the first metatarsophalangeal joint.

#### 3.9.4. Clinical Implications and Injection Strategy

The FHB is involved in spastic patterns such as:

- hallux claw foot deformity and hallux claw toe, which include hallux extension at the metatarsophalangeal joint and hallux flexion at the interphalangeal joint
- hallux valgus, together with the oblique and transverse heads of the adductor hallucis and the extensor hallucis longus [44,45]
- equinus/equinovarus, along with the soleus, tibialis posterior, flexor digitorum longus, flexor digitorum brevis, and flexor hallucis longus [17,27].

Local BoNT-A injections into these muscles improve plantar dorsiflexion range of motion and reduce spasticity of the leg muscles [27].

The region with the highest intramuscular nerve arborization of the FHB is located at 50–70% along the reference line connecting the middle-lowest point of the sole of the foot to the middle-lowest point of the great toe [44].

In our clinical practice, the preferred injection site corresponds to the area of maximal muscle thickness detected via musculoskeletal US, with the transducer placed transversely approximately 1–2 cm proximal to the first metatarsophalangeal joint.

### 3.10. Extensor Digitorum Longus (EDL)

#### 3.10.1. Overview

The extensor digitorum longus (EDL) is a muscle targeted in lower limb spasticity patterns characterized by resistance to passive movement during toe flexion (digits II–V) and plantar flexion at the ankle joint [9].

#### 3.10.2. Ultrasound Identification

In our clinical practice, the EDL can be identified using musculoskeletal ultrasound with the transducer placed transversely on the proximal third of the anterior aspect of the leg, in the lateral portion. Superficial to the fibular cortex, the EDL is visualized.

#### 3.10.3. Key Ultrasound Landmarks

**Figure 13:**
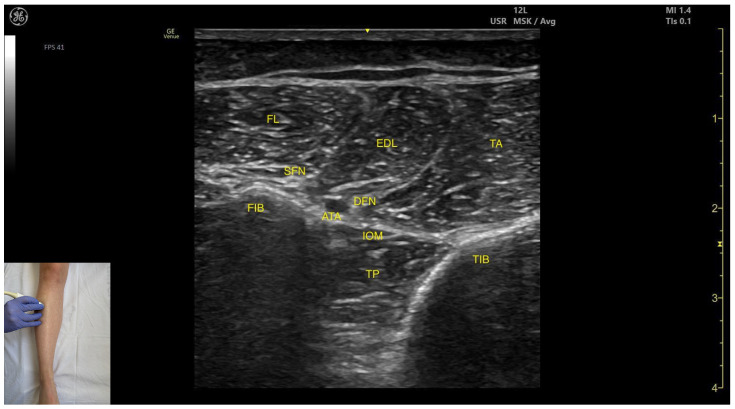
Ultrasound anatomy of the extensor digitorum longus: FIB—fibula; FL—fibularis longus; SFN—superficial fibular nerve; ATA—anterior tibial artery; DFN—deep fibular nerve; EDL—extensor digitorum longus; IOM—interosseous membrane; TP—tibialis posterior; TA—tibialis anterior; TIB—tibia.

The key ultrasound features include the following (Figure 13) [9,14]:

- Muscle position: It occupies the most posterior and lateral position within the anterior compartment of the leg. It lies superficially beneath the subcutaneous plane. Medial to it lies the tibialis anterior; lateral to it lies the fibularis longus.
- Neurovascular bundle: The superficial fibular nerve courses within the intermuscular fascia between the EDL and fibularis longus. Deep structures include the interosseous membrane, tibialis posterior muscle, deep fibular nerve, and the anterior tibial artery.
- External fascia: It presents a pronounced fascia that demarcates it from the superficial plane and adjacent muscles (tibialis anterior and tibialis posterior), aiding in safe BoNT-A injection.
- Dynamic evaluation: During dynamic evaluation, scanning toward the ankle joint, the EDL muscle thickness decreases as the EHL appears deep to it. Muscle contraction is visible during toe extension of digits II–V and dorsiflexion at the ankle joint.

#### 3.10.4. Clinical Implications and Injection Strategy

EDL plays a critical role in frontal-plane stabilization of the foot during gait. Reducing plantar-flexor spasticity while enhancing EDL function is therefore important to maintain normal gait mechanics [48]. Spasms of the extensor hallucis longus (EHL) and the EDL are associated with extension dystonia of the toes. These spasms produce local pain, difficulty with shoe wear, and gait impairment due to pain and instability during the stance phase [49]. Rucco et al. report that the EDL may contribute to the spastic “clawed-toes” pattern, often in conjunction with the extensor digitorum brevis [50].

The use of musculoskeletal ultrasound in approaching the EDL allows for the accurate targeting of the injection site and helps to avoid neurovascular injury. In our clinical practice, the preferred injection site corresponds to the area of maximal muscle thickness detected via musculoskeletal US. For the EDL, the injection site is identified with the transducer placed on the proximal third of the anterior aspect of the leg, in the lateral portion.

Table 1 captures the key tips and precautions for BoNT-A injections in each lower limb muscle.

## 4. Conclusions

This manuscript extends the Elias University Hospital (EUH) visual guide by concentrating on distal lower limb muscles—a crucial yet frequently underrepresented segment in spasticity management. Central to our injection strategy is the use of musculoskeletal ultrasound, which enables a systematic, evidence-informed approach to safely target ten muscles: the gastrocnemius, soleus, tibialis posterior, flexor hallucis longus, flexor digitorum longus, tibialis anterior, extensor hallucis longus, flexor digitorum brevis, flexor hallucis brevis and extensor digitorum longus. For each muscle, we present key anatomical landmarks, dynamic sonographic evaluation, zones of neural arborization, and tailored ultrasound-guided injection techniques grounded in both the literature and our extensive clinical experience at EUH.

We integrated sonographic images with dynamic evaluation and injection protocols and provided clinicians—regardless of experience level—with practical, reproducible tools to manage complex spastic patterns, including equinus, varus, claw foot, and toe deformities. This completes the continuum established in earlier installments focusing on upper limbs and proximal lower limbs, thereby offering a unified, standardized framework for ultrasound-guided botulinum toxin interventions across the entire limb.

Through this contribution, we aim to bridge the gap between descriptive anatomical atlases and real-world clinical practice, enhancing precision and confidence in BoNT-A administration and ultimately improving patient function, mobility, and quality of life.

## Figures and Tables

**Table 1:** Key safety considerations for ultrasound-guided botulinum toxin type A (BoNT-A) injections into distal lower limb muscles.

Muscle	Key Anatomical Landmarks	Nearby Structures at Risk	Ultrasound Technique Tip	Injection Precautions
Lateral Head of Gastrocnemius	~8 cm distal to the popliteal crease, on the lateral side	Medial sural cutaneous nerve (superficial), soleus and plantaris muscle/tendon if present (deep)	Transverse scan on the posterior aspect of the leg	Avoid medial sural cutaneous nerve injury
Medial Head of Gastrocnemius	~8 cm distal to the popliteal crease, on the medial side	Medial sural cutaneous nerve (superficial), soleus and plantaris muscle/tendon if present (deep)	Transverse scan on the posterior aspect of the leg	Avoid medial sural cutaneous nerve injury
Soleus	~8 cm distal to the popliteal crease on the midline	Gastrocnemius (superficial) and plantaris muscle/tendon (if present); posterior tibial artery, tibial veins, and the posterior tibial nerve (deep)	Transverse scan on the posterior aspect of the leg	Avoid neurovascular injury
Tibialis Posterior (anterior window)	~10 cm distal to the knee joint, in the lateral portion	Interosseous membrane, deep fibular nerve, anterior tibial artery, and anterior tibial vein (superficial); tibialis anterior (superficial and medial), extensor digitorum longus (superficial and lateral);	Transverse scan on the anterior aspect of the leg	Avoid neurovascular injury
Tibialis Posterior (medial window)	Distal third of the leg, in the medial portion	Flexor digitorum longus (superficial and medial), flexor hallucis longus (superficial and lateral); tibial nerve, posterior tibial artery, and posterior tibial vein (superficial); interosseous membrane (deep)	Transverse scan on the distal third of the leg	Avoid neurovascular injury
Tibialis Posterior (posterior window)	Distal third of the posterior leg	Flexor digitorum longus, tibial nerve, posterior tibial artery and posterior tibial vein (superficial and medial); flexor hallucis longus, fibular artery and vein (superficial and lateral); interosseous membrane (deep)	Transverse scan on the distal third of the leg	Avoid neurovascular injury
Flexor Hallucis Longus	Distal third of the posterior leg, in the lateral portion	Soleus (superficial); tibialis posterior, flexor digitorum longus (deep and medial); Interosseous membrane (deep)	Transverse scan on the distal third of the posterior leg	Confirm muscle position and relations
Flexor Digitorum Longus	Distal third of the posterior leg in the medial portion	Soleus (superficial); flexor hallucis longus, tibial nerve, posterior tibial artery, and posterior tibial veins (lateral); tibialis posterior, interosseous membrane (deep and lateral);	Transverse scan distal third of the posterior leg	Avoid neurovascular injury
Tibialis anterior	Proximal third of the anterior aspect of the leg, in the lateral portion	Extensor digitorum longus (lateral); Interosseous membrane, tibialis posterior (deep)	Transverse scan on proximal third of the anterior aspect of the leg	Confirm muscle position and relations
Extensor Hallucis Longus	Distal third of the anterior aspect of the leg, in lateral portion	Tendinous portion of the tibialis anterior (superficial and lateral), extensor digitorum longus (lateral); interosseous membrane and tibialis posterior (deep)	Transverse scan on distal third of the anterior aspect of the leg	Confirm muscle position and relations
Flexor Digitorum Brevis	Mid-plantar aspect of the foot	Plantar aponeurosis (superficial); abductor hallucis (medial); quadratus plantae (deep)	Transverse scan at mid-plantar aspect of the foot	Visualize flexor digitorum brevis as most superficial muscle and confirm muscle position and relations
Flexor Hallucis Brevis	~1–2 cm proximal to the first metatarsophalangeal joint	Abductor hallucis muscle and tendon (medial); flexor hallucis longus tendon (lateral); adductor hallucis (deep and medial)	Transverse scan ~1–2 cm proximal to the first metatarsophalangeal joint	Visualize flexor hallucis brevis as most superficial muscle and confirm muscle position and relations
Extensor Digitorum Longus	Proximal third of the anterior aspect of the leg, in the lateral portion	Tibialis anterior (medial); Fibularis longus (lateral); superficial fibular nerve, anterior tibial artery, deep fibular nerve (deep)	Transverse scan on proximal third of the anterior aspect of the leg	Visualize extensor digitorum longus as most superficial muscle and confirm muscle position and relations

## Data Availability

No new data were created or analyzed in this study.

## Supplementary Materials

The following supporting information can be downloaded at: https://doi.org/10.5281/zenodo.17316246 (accessed on 10 October 2025), Video S1: dynamic ultrasound evaluation of the flexor hallucis longus (FHL); Video S2: dynamic ultrasound evaluation of the flexor digitorum longus (FDL); Video S3: dynamic ultrasound evaluation of the extensor hallucis longus (EHL); Video S4: dynamic ultrasound evaluation of the flexor digitorum brevis (FDB).

## Abbreviations

The following abbreviations are used in this manuscript:

BoNT-A	Botulinum toxin type A
EUH	Elias University Hospital
Gastroc	Gastrocnemius
LG	Lateral head of gastrocnemius
MG	Medial head of gastrocnemius
SOL	Soleus
FIB	Fibula
TIB	Tibia
US	Ultrasound
MAS	Modified Ashworth Scale
PROM	Passive range of motion
GMFM	Gross Motor Function Measure
TP	Tibialis posterior
ATV	Anterior tibial vein
ATA	Anterior tibial artery
DFN	Deep fibular nerve
EDL	Extensor digitorum longus
TA	Tibialis anterior
IOM	Interosseous membrane
FDL	Flexor digitorum longus
FHL	Flexor hallucis longus
PTA	Posterior tibial artery
PTV	Posterior tibial vein
TN	Tibial nerve
FA	Fibular artery
FV	Fibular vein
EHL	Extensor hallucis longus
TAt	Tibialis anterior tendon
FDB	Flexor digitorum brevis
NAV	Navicular
AH	Adductor hallucis
QP	Quadratus plantae
CUB	Cuboid
FHB	Flexor hallucis brevis
AbH	Abductor hallucis
FHB-MH	Flexor hallucis brevis medial head
FHB-LH	Flexor hallucis brevis lateral head
FL	Fibularis longus
SFN	Superficial fibular nerve
MSCN	Medial sural cutaneous nerve
EMG	Electromyography
FDB2	Flexor digitorum brevis to second toe
FDB3	Flexor digitorum brevis to third toe
FDB4	Flexor digitorum brevis to fourth toe
FDB5	Flexor digitorum brevis to fifth toe
